# Effect of pulmonary rehabilitation in patients with chronic obstructive pulmonary disease: a systematic review and meta-analysis of randomized controlled trials

**DOI:** 10.1080/07853890.2021.1999494

**Published:** 2022-01-17

**Authors:** Hong Zhang, Dandan Hu, Yikai Xu, Lixia Wu, Liming Lou

**Affiliations:** Department of Respiratory Medicine, The Third Affiliated Hospital of Zhejiang Chinese Medical University, Hangzhou, China

**Keywords:** Pulmonary rehabilitation, chronic obstructive pulmonary disease, randomized controlled trials, systematic review, meta-analysis

## Abstract

**Objective:**

The present systematic review and meta-analysis of randomized clinical trials (RCTs) aimed to investigate the effects of pulmonary rehabilitation in individuals with chronic obstructive pulmonary disease (COPD).

**Methods:**

The RCTs of pulmonary rehabilitation programs published between 1999 and 2021 were retrieved from electronic databases (PubMed, Cochrane Library, and Embase). Two reviewers independently assessed the topical relevance and trial quality and extracted data for meta-analysis using the Stata software version 14.0.

**Results:**

A total of 39 trials involving 2,397 participants with COPD were evaluated. We found that patients who received pulmonary rehabilitation program had significant improvement in the 6-min walk test (6MWT), St. George Respiratory Questionnaire score, and the modified British Medical Research Council score as compared to those who received usual care. Yoga and Tai Chi showed significant improvement in the forced expiratory volume (FEV1)% in 1 s predicted value. However, no significant difference was detected in the modified Borg score, forced vital capacity (FVC), and FEV1/FVC predicted value between the pulmonary rehabilitation and usual care groups.

**Conclusion:**

Yoga and Tai Chi showed a significant improvement in the FEV1% predicted value. Also, pulmonary rehabilitation program improved the exercise capacity, the quality of life, and dyspnoea in patients with COPD.

**Key messages**
- A total of 39 trials involving 2,397 participants with COPD were evaluated.
- We found that patients who received pulmonary rehabilitation program had significant improvement in the 6MWT, St. George Respiratory Questionnaire score, and the modified British Medical Research Council score as compared to those who received usual care.
- Yoga and Tai Chi showed significant improvement in the FEV1% predicted value.
- No significant difference was detected in the modified Borg score, FVC, and FEV1/FVC predicted value between the pulmonary rehabilitation and usual care groups.

## Introduction

Chronic obstructive pulmonary disease (COPD) is a common chronic disease characterized by persistent respiratory symptoms and airflow limitation [1]. COPD is a progressive and debilitating respiratory disease, leading to a severe burden on the individual and society. It is the only chronic disease with increasing morbidity and mortality [2]. The World Health Organisation (WHO) predicts that by 2030, COPD will become the third leading cause of deaths worldwide [3]. China has the most significant number of COPD patients in the world: about 99 million, with an 8.6% prevalence. The number of deaths each year exceeds 900,000 [4,5].

In COPD therapy, pulmonary rehabilitation is regarded as the hallmark of treatment in all patients [6]. Typically, the pulmonary rehabilitation program is implemented by multidisciplinary teams in the outpatient department, including exercise training, education, nutritional supplement, and psychosocial support. Compared to the traditional community care, pulmonary rehabilitation reduces dyspnoea and fatigue and improves exercise endurance and many areas of health-related quality of life (HRQOL) [7–9].

The main purpose of pulmonary rehabilitation training is to formulate a corresponding pulmonary rehabilitation plan according to the actual situation of the patient, thereby improving the patient’s quality of life and exercise endurance and the symptoms of dyspnoea [10]. In addition, this personalized treatment plan can reduce complications, enhance endurance, social participation, and reduce medical budget [11]. The pulmonary rehabilitation of patients with COPD mainly includes functional exercise, education, oxygen therapy, nutritional support, and psychotherapy [12]. As early as the 1970s, Gimenez et al. [13] conducted a 10-year follow-up study on the pulmonary rehabilitation of patients with COPD. Several randomized controlled trials (RCTs) have investigated the effect of pulmonary rehabilitation for COPD patients, and no consistent outcomes have been reported [14–17]. In addition, a large number of studies [18–31] have been carried out on the influence of mind–body exercise (Tai Chi, Yoga) on COPD, however, the previous meta-analysis [7,32–34] did not include them. Thus, this meta-analysis was conducted to assess the efficacy of pulmonary rehabilitation (including Tai Chi and Yoga) in patients with COPD.

## Materials and methods

### Search strategy

The databases, including PubMed, Embase, and Cochrane Library, were queried using the keywords “chronic obstructive pulmonary disease/COPD”, “pulmonary rehabilitation”, “exercise training”, “Yoga”, “Tai Chi”, and “randomized controlled trial/RCT”. The search was updated until August 2021. The detailed search strategy is shown in Supplementary Table S1. No restrictions were applied to the language, and cross-references and reviews were assessed to gather all the eligible publications.

### Inclusion and exclusion criteria

Studies were included if the following criteria were fulfilled [1]: included patients had a clinical diagnosis of COPD [2]; study design involved an RCT [3]; studies having at least two groups: one group receiving pulmonary rehabilitation and another group receiving usual care [4]; participants received pulmonary rehabilitation programs based on Yoga, Tai Chi, and conventional physical exercises, such as walking, jogging, swimming, and cycling. COPD patients diagnosed according to the Global Obstructive Lung Disease Initiative criteria (Global Strategy for the Diagnosis, Management and Prevention of COPD). The following studies were excluded [1]: reviews, editorials, conference abstracts, letters, and case reports [2]; duplicate publications [3]; basic research or animal studies [4]; studies without sufficient data.

### Data extraction and quality assessment

All available data were extracted independently by two researchers according to the inclusion criteria. Any differences were resolved through discussions with the third author. The following data were extracted from each article: first author’s name, year of publication, research design, country, gender, mean age, mean forced expiratory volume in one second (FEV1), sample size, follow-up time, and outcomes assessed. Also, we assessed the risk of bias using the Cochrane Collaboration tool for risk of bias assessment [35].

### Data synthesis and analysis

The weighted mean difference (WMD) and 95% confidence interval (CI) were calculated for continuous data. Then, the data were combined according to random-effects (DerSimonian and Laird’s method) or fixed-effects model depending on the significance of the *I*^2^ statistic (*I*^2^ > 50% was considered statistically significant). If the heterogeneity was significant, the random-effects model was adopted, otherwise fixed-effects model was adopted. The sequential removal of each study for sensitivity analysis was carried out to assess the relative impact of each study comprehensively. Egger’s linear regression test and Begg’s test are used to demonstrate the publication bias; if *p* < .05, we used the trim and fill method for corrections [36]. The statistical analysis was performed using the Stata software version 14.0 (Stata, TX, USA), and all tests were double-sided.

## Results

### Study properties

**Figure 1.**
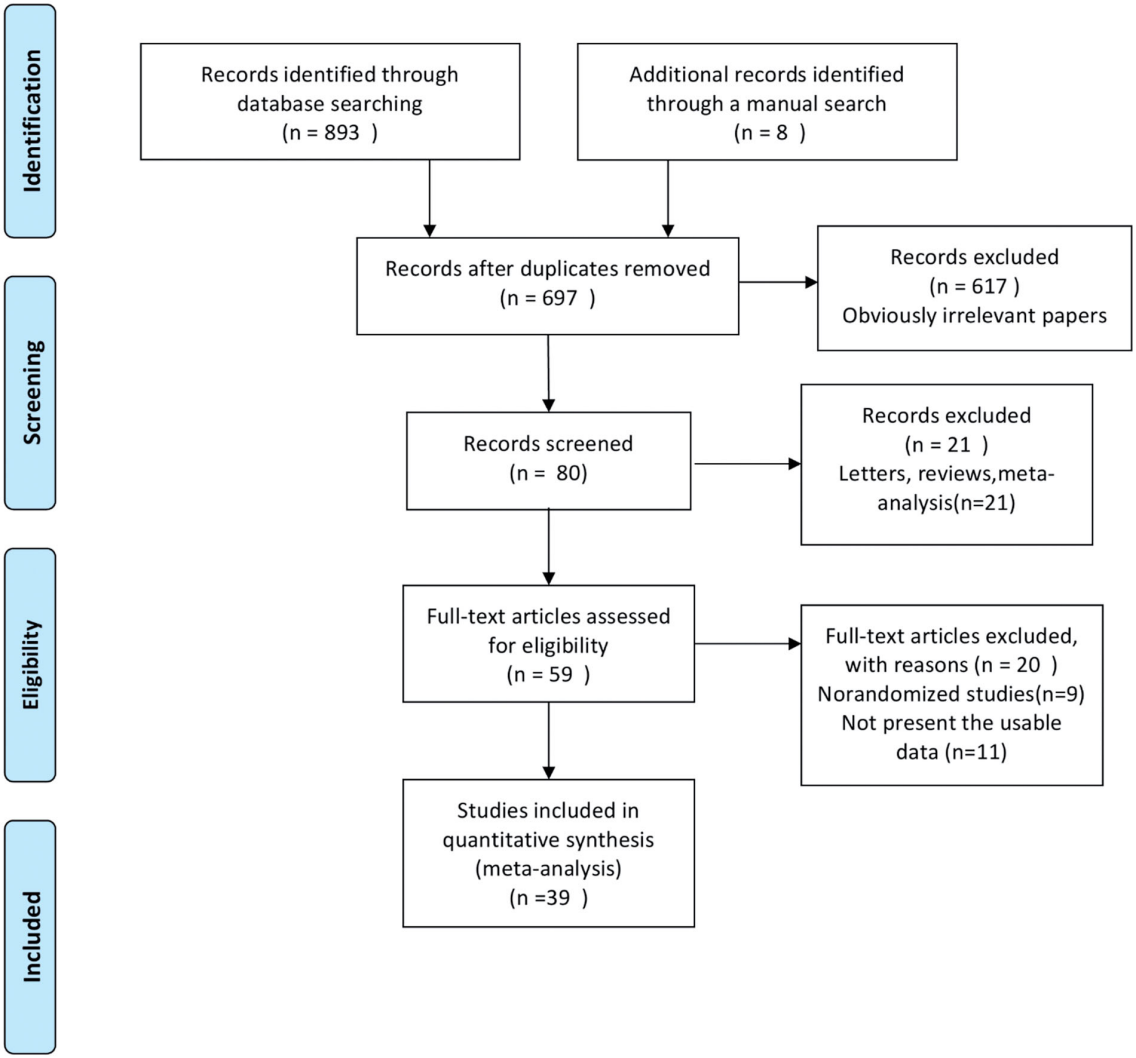
Flow diagram for the identification of the studies.

**Figure 2.**
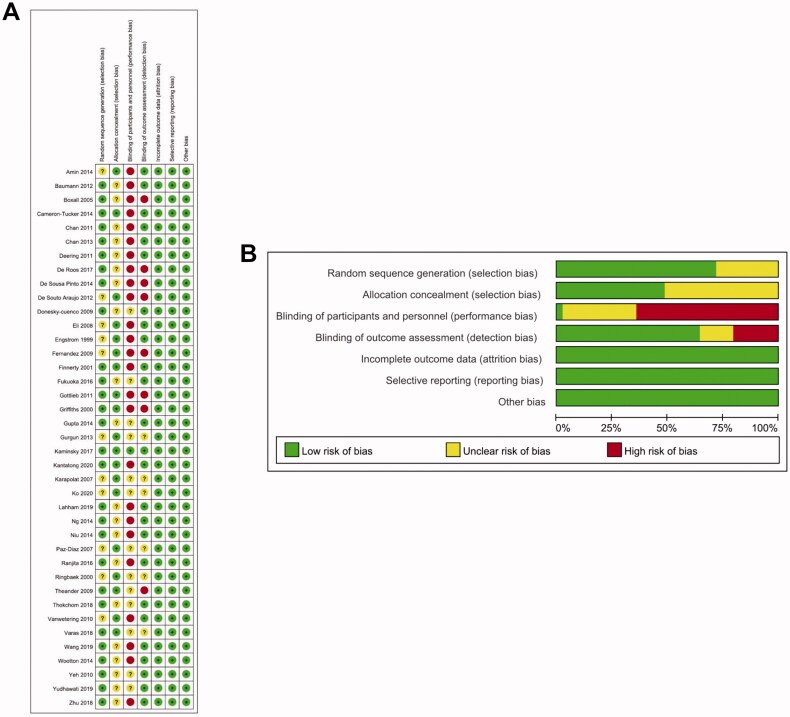
Risk of bias assessment for the randomized trials included in the meta-analysis. (A) Risk of bias summary; (B) Risk of bias graph. *Symbols*. (+): low risk of bias; (?): unclear risk of bias; (–): high risk of bias.

Overall, 893 articles were retrieved from four databases, and 8 articles were identified *via* manual search. The duplicates were excluded, and after screening the abstracts of the remaining articles, 59 full-text articles were obtained for further review (Figure 1). Based on our selection criteria, 20 studies were further excluded, and finally, 39 studies were included in this meta-analysis [14–31,37–57] (Figure 1). The baseline characteristics of included studies are listed in Table 1. The studies included in the meta-analysis have been conducted in China (19.4%, 7/36), the USA (13.9%, 5/36), Australia (11.1%, 4/36), Turkey (8.3%, 3/36), India (8.3%, 3/36), Sweden (5.6%, 2/36), the UK (5.6%, 2/36), Denmark (5.6%, 2/36), Spain (5.6%, 2/36), Netherlands (5.6%, 2/36), Brazil (5.6%, 2/36), Ireland (2.8%, 1/36), Japan (2.8%, 1/36), Indonesia (5.6%, 1/36), Thailand (2.8%, 1/36), and Germany (2.8%, 1/36). These 36 studies involved a total of 2,397 patients, and the sample size of each study was 8–200 patients. The articles included in the present study were in English and published between 1999 and 2020. The Cochrane Collaboration tool for assessing the risk of bias (Figure 2) showed that poor scores were obtained in the performance and detection bias due to the nature of the intervention as it was not possible to blind the subjects to their allocation.

**Table 1.** Characteristics of the studies included in this meta-analysis.

Authors/year of publication	Country	Mean age (years)	Male (%) PR/Con	Mean FEV1 (% or L) PR/Con	Type of study	Intervention		Follow-up
						PR	Con	
Engstrom/1999 [37]	Sweden	PR: 66 ± 5.4	53.8/50	30.7/34.1	RCT	26	24	12M
		Con: 66.8 ± 5.4						
Griffiths/2000 [14]	UK	PR: 68.2 ± 8.2	61.6/58.4	39.7/39.4	RCT	99	101	12M
		Con: 68.3 ± 8.1						
Ringbaek/2000 [38]	Denmark	PR:61.8 ± 6.8	4.17/28.6	49.5/44.3	RCT	24	21	2M
		Con: 64.6 ± 7.7						
Finnerty/2001 [39]	UK	PR:70.4 ± 8.0	69.4/65.5	41.2/41.2	RCT	36	29	6M
		Con: 68.4 ± 10.4						
Boxall/2005 [15]	Australia	PR:77.6 ± 7.6	47.8/65.2	40.5/37.7	RCT	23	23	3M
		Con: 75.8 ± 8.1						
Karapolat/2007 [40]	Turkey	PR: 65.1 ± 9.4	81.5/95.5	54.8/55	RCT	27	22	3M
		Con: 66.6 ± 8.4						
Paz-Diaz/2007 [41]	USA	PR: 67 ± 5	60/85.7	34/30	RCT	10	14	2M
		Con: 62 ± 7						
Eli/2008 [42]	Turkey	PR: 59.67 ± 8.6	15.4/15.4	47.77/46.28	RCT	39	39	3M
		Con: 58.08 ± 11.45						
Donesky-cuenco/2009 [25]	USA	PR: 72.2 ± 6.5	28.6/26.7	51.2/44.4	RCT	14	15	3M
		Con: 67.7 ± 11.5						
Fernandez/2009 [43]	Spain	PR: 66 ± 8	NA	33/38	RCT	27	14	12M
		Con: 70 ± 5						
Theander/2009 [44]	Sweden	PR: 66 ± 2	25/71.4	35.1/32.3	RCT	12	14	3M
		Con: 64 ± 2						
Vanwetering/2010 [45]	Netherlands	64 ± 8.7	61.5	54.7	RCT	16	14	4M
Yeh/2010 [23]	USA	PR: 65 ± 2	60/60	53/47	RCT	5	5	3M
		Con: 66 ± 6						
Chan/2011 [46]	China	PR: 73.6 ± 7.5	88/87	0.91/0.89	RCT	69	67	3M
		Con: 73.6 ± 7.4						
Deering/2011 [47]	Ireland	PR: 67.7 ± 5.3	NA	48.5/45.8	RCT	25	19	3M
		Con: 68.6 ± 5.5						
Gottlieb/2011 [16]	Denmark	PR: 74.1	31.8/35	64.27/67.05	RCT	22	20	18M
		Con: 73.2						
Baumann/2012 [48]	Germany	PR: 63 ± 11	62.2/54.5	47/45	RCT	37	44	6M
		Con: 65 ± 8						
De Souto Araujo/2012 [49]	Brazil	PR: 56.9 ± 7.9	61.5/72.7	39.2/45.1	RCT	13	11	2M
		Con: 71.1 ± 10.1						
Chan/2013 [18]	China	PR: 71.7 ± 8.2	99/87	0.89/0.89	RCT	70	67	6M
		Con: 73.6 ± 7.4						
Gurgun/2013 [17]	Turkey	PR: 66.8 ± 9.6	100/100	41.9/39.3	RCT	15	16	2M
		Con: 67.8 ± 6.6						
Amin/2014 [50]	USA	PR: 66.8 ± 8.1	33/60	63.6/60.8	RCT	9	10	3M
		Con: 72 ± 10.1						
Cameron-Tucker/2014 [52]	Australia	PR: 64.5 ± 9.13	53/54	NA	RCT	43	41	1.5M
		Con: 67.1 ± 9.41						
De Sousa Pinto/2014 [54]	Brazil	PR: 68.9 ± 9.2	95.7/94.4	33.5/34.5	RCT	23	18	3M
		Con: 71.9 ± 7.6						
Gupta/2014 [27]	India	PR: 52.5 ± 3.9	96/96	51.1/49.6	RCT	25	25	3M
		Con: 52 ± 4.1						
Ng/2014 [20]	China	PR: 74.16 ± 6.46	93.6/88.8	1.1/1.23	RCT	94	98	6M
		Con: 74.13 ± 6.81						
Niu/2014 [21]	China	PR: 59.7 ± 2.76	95/90	41.9/43.7	RCT	20	20	6M
		Con: 61.3 ± 2.89						
Wootton/2014 [57]	Australia	PR: 69 ± 8	58.9/58.3	43/43	RCT	95	48	2M
		Con: 68 ± 9						
Fukuoka/2016 [26]	Japan	PR: 74.6 ± 6.7	100/66.7	0.93/1.2	RCT	5	3	0.5M
		Con: 77 ± 7						
Ranjita/2016 [29]	India	PR: 53.69 ± 5.66	NA	NA	RCT	36	36	3M
		Con: 54.41 ± 5.4						
De Roos/2017 [53]	Netherlands	PR: 69.4 ± 9.7	31/38	68/65	RCT	26	26	2.5M
		Con: 71 ± 9.4						
Kaminsky/2017 [28]	USA	PR: 68 ± 7	33/45	43/42	RCT	21	22	3M
		Con: 68 ± 9						
Thokchom/2018 [30]	India	PR: 57.8 ± 2.68	76.2/80	1.24/1.22	RCT	21	20	3M
		Con: 60.65 ± 1.84						
Varas/2018 [56]	Spain	PR: 69.5 ± 7.4	85.7/68.4	45.8/52.3	RCT	21	19	12M
		Con: 64.8 ± 9.1						
Zhu/2018 [24]	China	PR: 67.87 ± 5.22	93/97	35.11/40.77	RCT	30	30	9M
		Con: 68.1 ± 6.57						
Lahham/2019 [51]	Australia	PR: 68 ± 9	58.6/58.6	0.9/0.92	RCT	29	29	6M
		Con: 67 ± 10						
Wang/2019 [22]	China	PR: 67.83 ± 5.32	88.5/87.5	55.46/62.55	RCT	26	24	3M
		Con: 67.86 ± 5.98						
Yudhawati/2019 [31]	Indonesia	PR: 64.4 ± 10.4	NA	43.53/40.87	RCT	15	15	3M
		Con: 65.33 ± 8.1						
Kantatong/2020 [19]	Thailand	PR: 69.68 ± 7.67	60/76	68.21/68.37	RCT	25	25	3M
		Con: 67.48 ± 10.17						
Ko/2020 [55]	China	PR:76 ± 8	99/96	49/46	RCT	68	68	12M
		Con: 74 ± 7						

PR: pulmonary rehabilitation; Con: control; RCT: randomized controlled trials; FEV1: forced expiratory volume in one second; L: litre; Y: years; M: months; NA: not available.

### Meta-analysis outcome

#### 6-Min walk test (6MWT)

**Figure 3.**
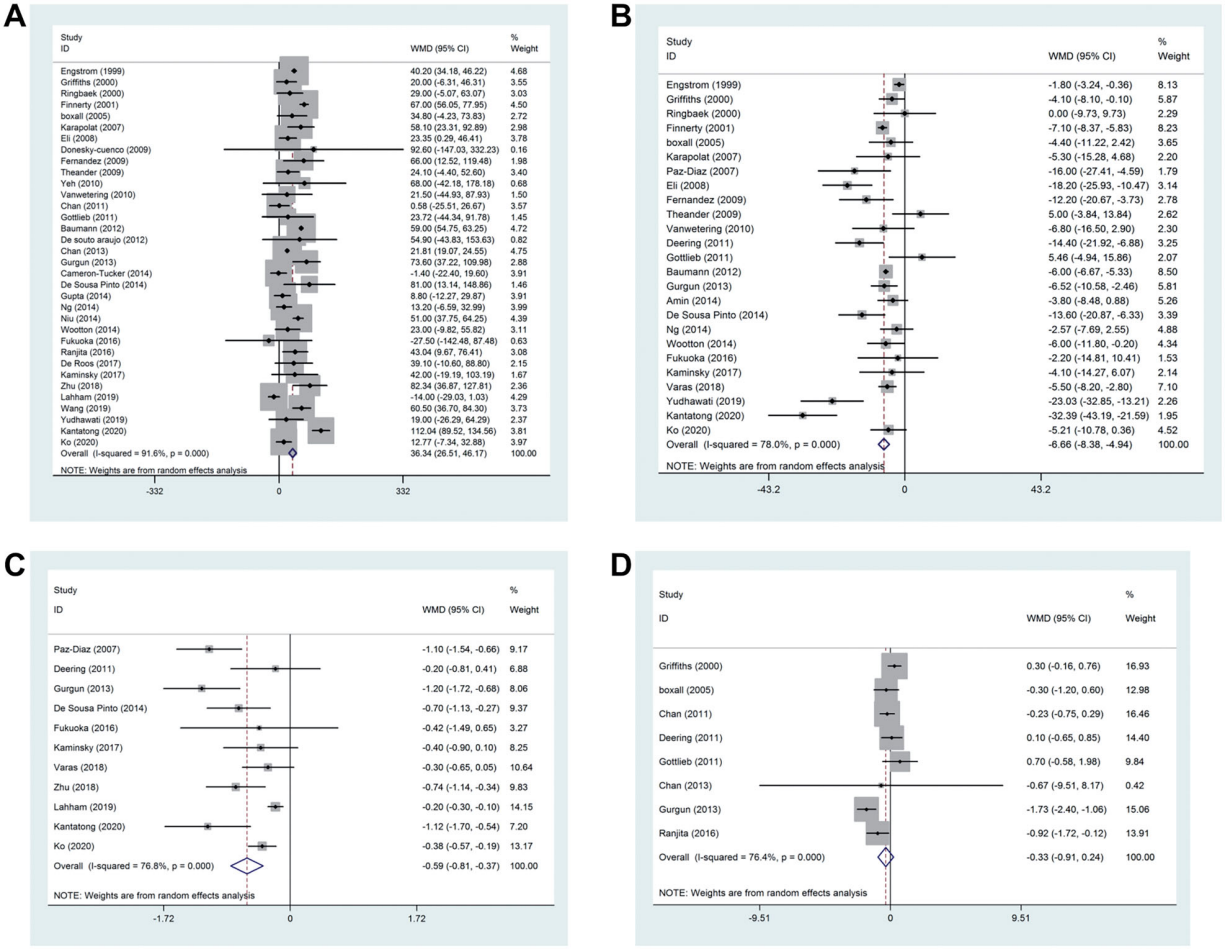
Effect of pulmonary rehabilitation in individuals with COPD. (A) 6MWT; (B) SGRQ score; (C) MRC; (D) Borg score.

The changes in the exercise capacity from baseline were measured using 6MWT in 34 studies. The 6MWT distance was significantly improved (weighted mean difference (WMD), 36.34; 95% confidence interval (CI): 26.51–46.17; *p* < .001; *I^2^* = 91.6%) in the pulmonary rehabilitation group compared to the control group (Figure 3(A)).

#### St george respiratory questionnaire (SGRQ) score

The SGRQ score was reported in 25 studies. Pulmonary rehabilitation showed a significant improvement in the quality of life according to the altered SGRQ total score (WMD, −6.66; 95% CI: −8.38 to −4.94; *p* < .001; *I^2^* = 78%) (Figure 3(B)).

#### Modified british medical research council (MRC)

Dyspnoea was measured using the modified British MRC questionnaire in 11 studies. Pulmonary rehabilitation showed significant changes in the MRC (WMD, −0.59; 95% CI: −0.81 to −0.37; *p* < .001; *I^2^* = 76.8%) (Figure 3(C)).

#### Modified borg score

Borg score was reported in 8 studies, and no significant difference (WMD, −0.33; 95% CI: −0.91–0.24; *p* < .001; *I^2^* = 76.4%) was detected between the pulmonary rehabilitation group and the control group (Figure 3(D)).

#### Forced expiratory volume in one second (FEV1) percentage of predicted normal value

**Figure 4.**
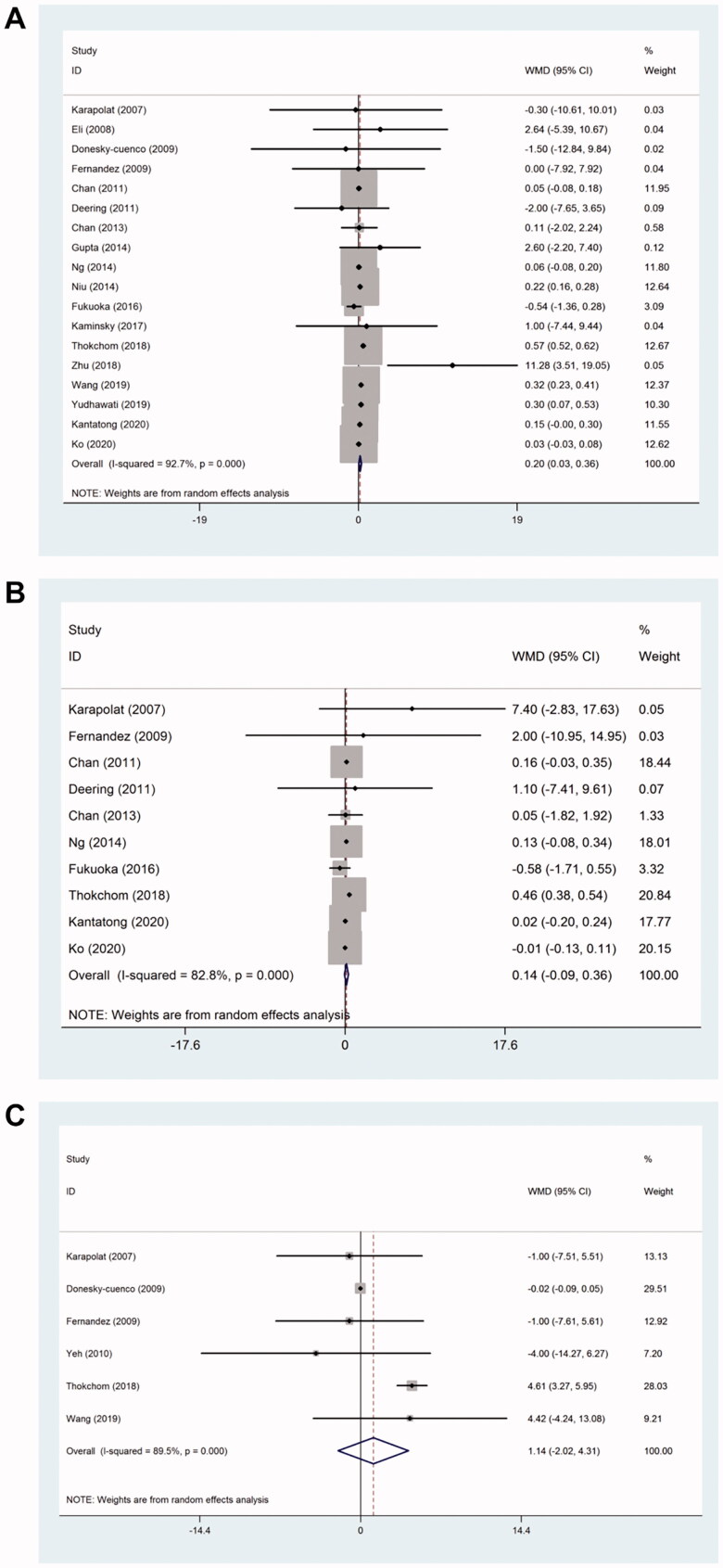
Effect of pulmonary rehabilitation on lung function in individuals with COPD. (A) FEV1%; (B) FVC%; (C) FEV1/FVC%.

FEV1% predicted value was reported in 18 studies. Pulmonary rehabilitation showed significant changes in the FEV1% predicted value (WMD, 0.20; 95% CI: 0.03–0.36; *p* < .001; *I^2^* = 92.7%) (Figure 4(A)).

#### FVC percentage of predicted normal value

FVC% predicted value was reported in 10 studies, and did not differ significantly (WMD, 0.14; 95% CI: −0.09–0.36; *p* < .001; *I^2^* = 82.8%) between the pulmonary rehabilitation group and the control group (Figure 4(B)).

#### FEV1/FVC percentage of predicted normal value

*FEV1/FVC* % predicted value was reported in 6 studies. Interestingly, no significant difference (WMD, 1.14; 95% CI: −2.02–4.31; *p* < .001; *I^2^* = 89.5%) was observed between the pulmonary rehabilitation and the control groups (Figure 4(C)).

### Sensitivity analysis

Sensitivity analysis was performed to examine whether the removal of each study would cause a significant change in the overall trend. However, the results were not altered after the sequential removal of each study, suggesting the reliability and stability of the results in this meta-analysis (Supplementary Figure S1).

### Subgroup analysis

**Figure 5.**
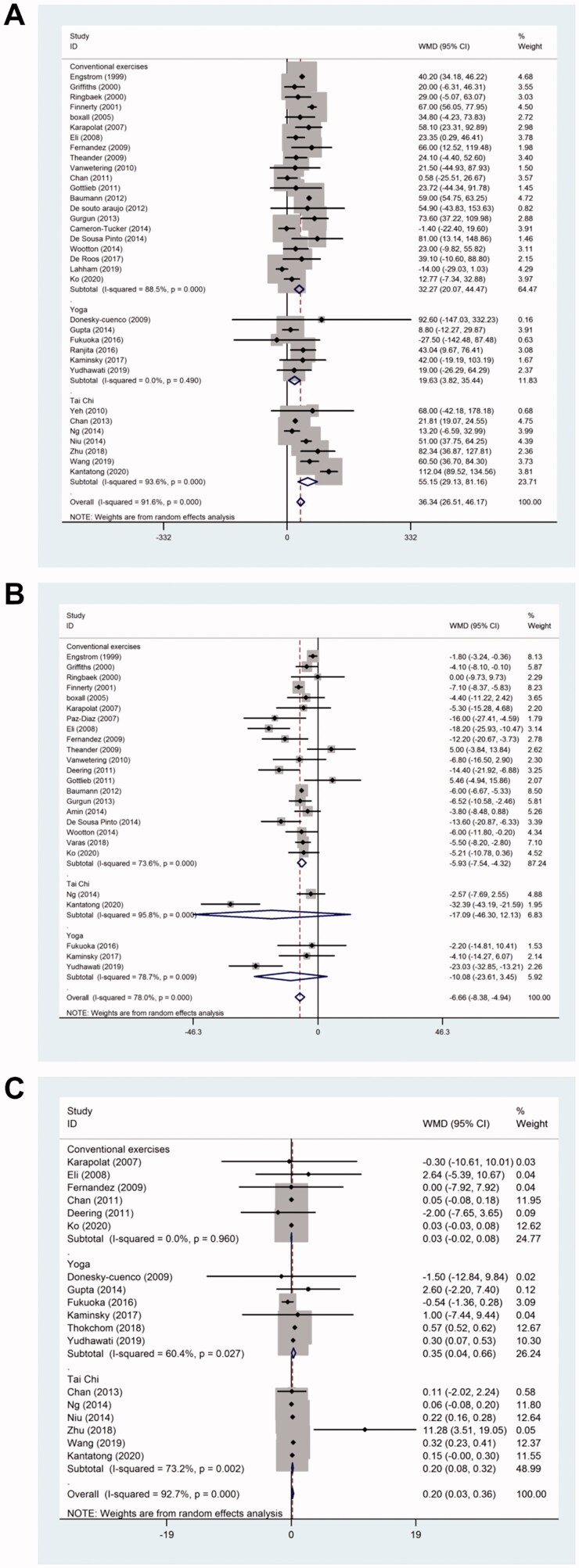
Subgroup analysis of the effect of pulmonary rehabilitation in individuals with COPD. (A) 6MWT; (B) SGRQ score; (C) FEV1.

The subgroup analysis was performed based on various rehabilitation measures since heterogeneity between the studies was observed in the overall comparisons. For the 6MWT, a significant improvement was observed in the Yoga (WMD, 19.63; 95% CI: 3.82–35.44; *p* = .49; *I^2^* = 0), Tai Chi (WMD, 55.15; 95% CI: 29.13–81.16; *p* < .001; *I^2^* = 93.6%), and the conventional exercises (WMD, 32.27; 95% CI: 20.07–44.47; *p* < .001; *I^2^* = 88.5%) group (Figure 5(A)). For the SGRQ score, a significant improvement was observed in the conventional exercises group (WMD, −5.93; 95% CI: −7.54 to −4.32; *p* < .001; *I^2^* = 73.6%) (Figure 5(B)) but not in the Yoga and Tai Chi groups. Furthermore, a significant improvement was observed in FEV1 values in the Yoga (WMD, 0.35; 95% CI: 0.04–0.66; *p* = .027; *I^2^* = 60.4%) and Tai Chi groups (WMD, 0.20; 95% CI: 0.08–0.32; *p* = 0.002; *I^2^* = 73.2%) (Figure 5(C)) but none was detected in the conventional exercises group.

### Publication bias

**Figure 6.**
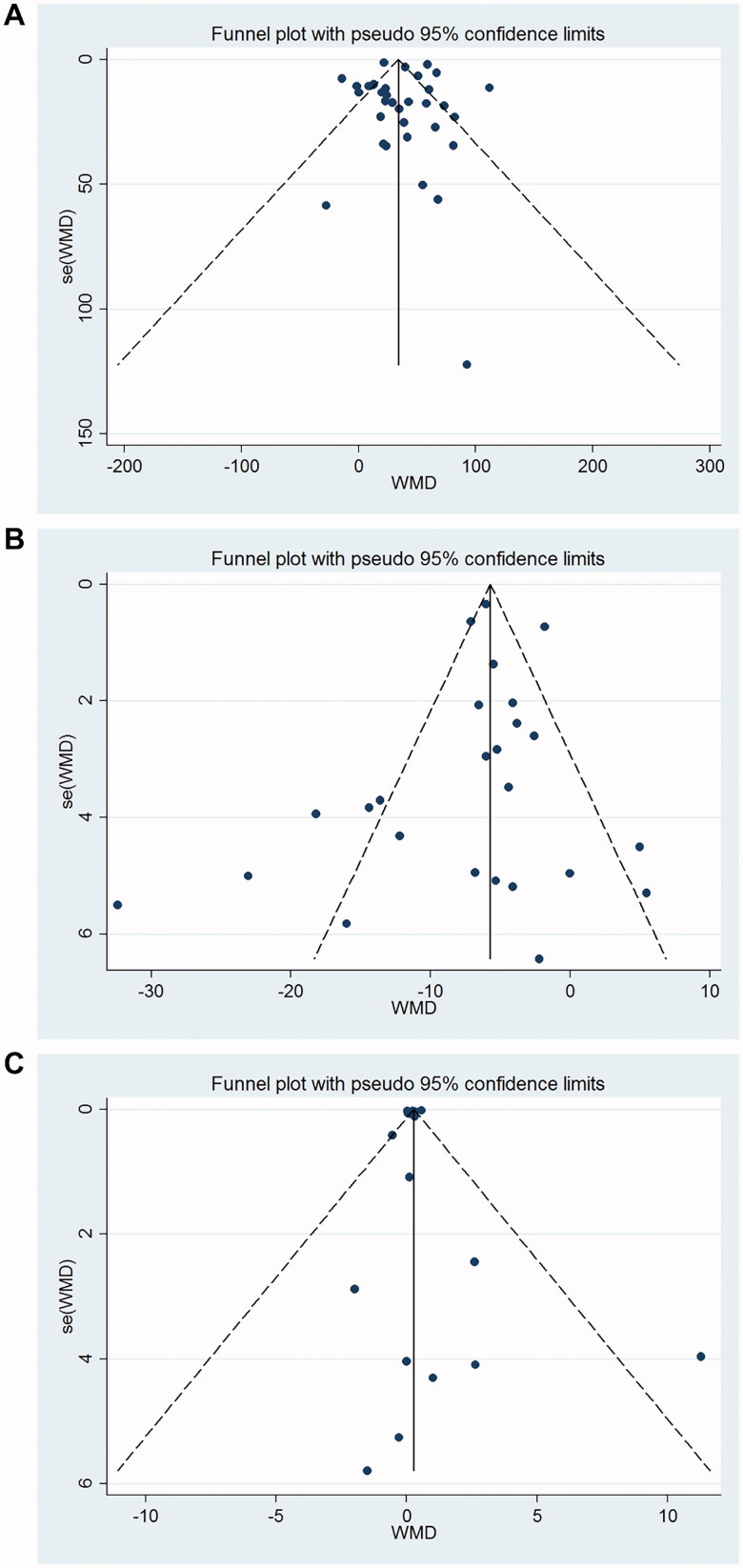
Funnel plot for publication bias test. Each point represents a separate study for the indicated association. (A) 6MWT; (B) SGRQ score; (C) FEV1.

Egger’s linear regression test and Begg’s test showed no publication bias for 6MWD (Begg’s test *p* = 0.343; Egger’s test *p* = .63) (Figure 6(A)), SGRQ (Begg’s test *p* = 0.624; Egger’s test *p* = .389) (Figure 6(B)), and FEV1 (Begg’s test *p* = .289; Egger’s test *p* = .746) (Figure 6(C)).

## Discussion

A comprehensive search was performed for RCTs that evaluated the efficacy of pulmonary rehabilitation in patients with COPD, and finally, 39 trials involving 2,397 COPD patients met our inclusion criteria. This meta-analysis showed that Yoga and Tai Chi significantly improved the FEV1% predicted value. Patients who received the pulmonary rehabilitation program had a marked improvement in the exercise capacity, quality of life, and dyspnoea compared to those who received usual care. Our study verifies known effects of the traditional PR, but that Yoga and Tai Chi can have significant improvement and may have benefit in FEV1 which PR does not do.

Pulmonary rehabilitation was first defined by the American College of Chest Physicians Committee in 1974. It is a proactive method that minimizes COPD symptoms, improves HRQOL, and increases physical and emotional participation in daily life [6,58]. In the latest update, pulmonary rehabilitation is defined as “Comprehensive interventions based on thorough patient assessment, followed by patient-tailored treatment, including but not limited to exercise training, education, and behavioural changes, aimed at improving the physical and psychological well-being of patients with chronic respiratory disease and promoting long-term adherence to health-promoting behaviors” [59]. In order to promote physical and mental health, mind-body exercise has attracted significant attention in the scientific community. The mind-body exercise focuses on mind, body, psychology, and behaviour, including breathing, physical exercise, and meditation [60,61]. It is characterized by gentle and slow movements and body and breathing coordination, represented by Chinese traditional sports Tai Chi and Indian Yoga [62,63]. Mind-body exercise (Tai Chi and Yoga) is easy to learn and practice compared to other forms of exercise, with minimal requirement of equipment and venue [64].

The efficacy of pulmonary rehabilitation in patients with COPD has been investigated by previous meta-analyses. To the best of our knowledge, this is the largest meta-analysis to investigate the efficacy of pulmonary rehabilitation in patients with COPD, encompassing 2,397 patients in 39 RCTs. Recently, Dong et al. [65] conducted a comprehensive meta-analysis about the efficacy of pulmonary rehabilitation in patients with COPD. In the present study, we included newer RCTs, involving more COPD patients than that in the study by Dong et al. and performed a detailed analysis with respect to the exercise capacity, the quality of life, dyspnoea, and lung function.

Pulmonary rehabilitation is a healthy approach for patients with COPD. The main purpose of pulmonary rehabilitation training is to formulate a corresponding program according to the actual situation of the patient to improve the patient’s quality of life and exercise endurance and improve the symptoms of dyspnoea [66]. In clinical practice, 6MWT is often used to assess the changes in the pulmonary functional ability of COPD patients after pulmonary rehabilitation [67]. In this study, pulmonary rehabilitation group showed significantly increased 6MWT distance following intervention when compared individually to the control group, in accordance with the relevant literature [17,40,43]. The evaluation of HRQOL is also a critical issue that needs to be considered while formulating a treatment strategy and evaluating the results. Furthermore, pulmonary rehabilitation has a significant improvement in the quality of life according to the change in SGRQ total score. It is well-documented that individuals with COPD have impaired HRQOL [58,68]. Previous studies demonstrated that SGRQ scores in all areas were improved in COPD patients after pulmonary rehabilitation [69,70].

In COPD Dyspnoea is one of the main respiratory symptoms. At present, several scales can be used to classify and characterize dyspnoea: clinical scale (such as MRC) and psychophysical scale (such as Borg scale) are the most commonly used scales in daily clinical practice [71]. However, from a methodological point of view, special attention should be paid when using Borg scale. Operators must assess the patient's emotional orientation beforehand, make sure they know all the information they need to complete the scale, and that the symptom score is related to feelings, i.e. not judged or corrected. Dyspnoea, as a limiting factor in physical activity, is a chief complaint in patients with COPD. The present study showed that pulmonary rehabilitation significantly altered MRC, as described previously [17,41,54].

Nevertheless, the present meta-analysis has some limitations. First, we did not look for unpublished literature, which is not in line with Cochrane’s method. Second, the heterogeneity of different types of pulmonary rehabilitation programs, their intensity, duration, and quality of learning were prepared for intervention. These factors are incommensurable in the majority of the experiments. Furthermore, the duration of treatment regimens was inconsistent in different studies. Also, although we have summarized the results of all trials, the sample size in the current review might not be sufficient to exclude any significant experimental errors. Finally, although a series of outcome measures are used, the impact of the smallest clinically significant difference based on each indicator may not be reflected.

## Conclusions

Despite the limitations, this meta-analysis confirmed that Yoga and Tai Chi have significantly improved the FEV1% predicted value. The pulmonary rehabilitation program improves the exercise capacity, the quality of life, and dyspnoea in patients with COPD. However, additional studies on large datasets and well-designed models are required to substantiate these findings.

## Supplementary Material

Supplemental Material

## Data Availability

The data that support the findings of this study are available from the corresponding author upon reasonable request.
