# Supplementary figures and images for: Effect of pulmonary rehabilitation in patients with chronic obstructive pulmonary disease: a systematic review and meta-analysis of randomized controlled trials
Source: Ann Med. 2022 Jan 17;54(1):262–73. doi: 10.1080/07853890.2021.1999494 (PMC8765243; doi:10.1080/07853890.2021.1999494)

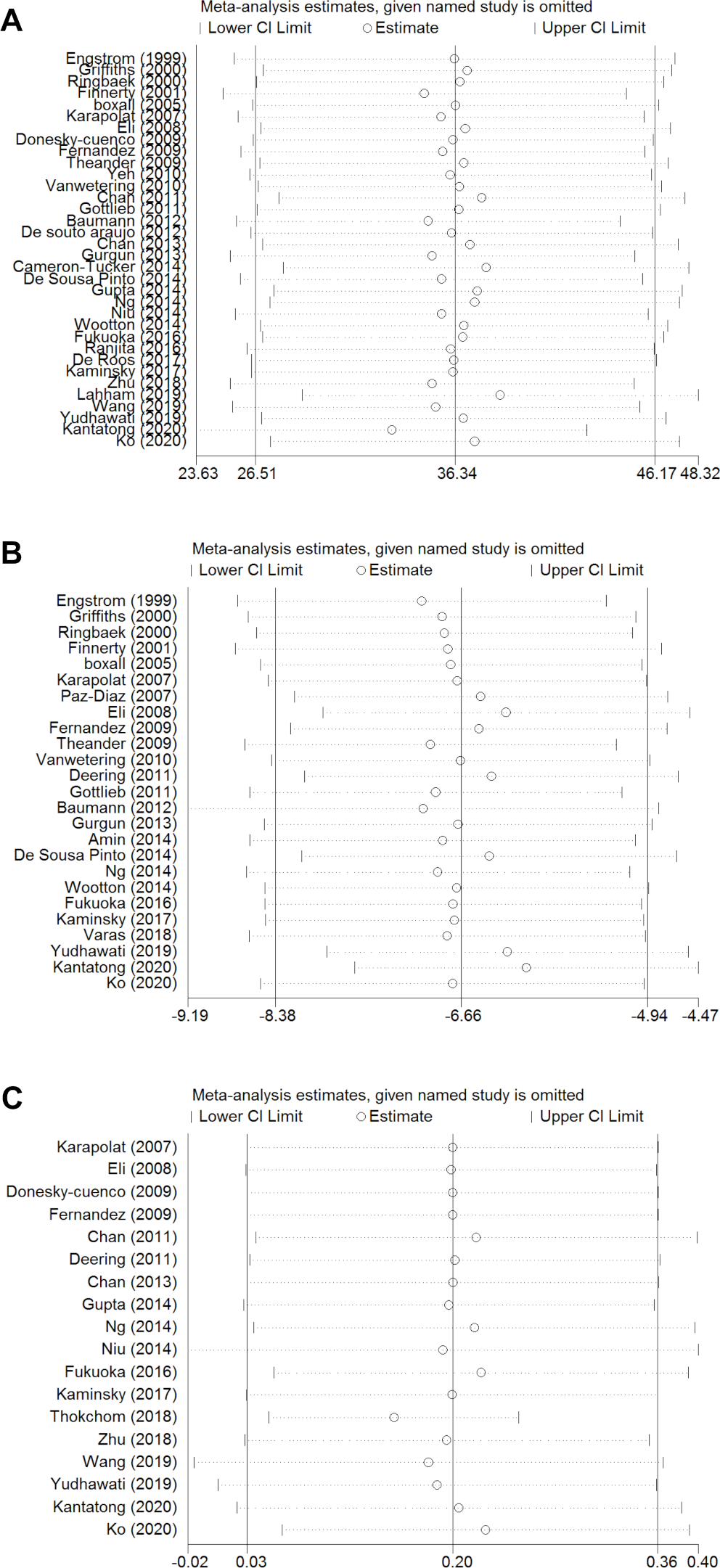

Supplement: Supplemental Material [file IANN_A_1999494_SM9330.zip › Supplemental files/Figure S1.tif]
