# Supplementary material for: Effect of pulmonary rehabilitation in patients with chronic obstructive pulmonary disease: a systematic review and meta-analysis of randomized controlled trials
Source: Ann Med. 2022 Jan 17;54(1):262–73. doi: 10.1080/07853890.2021.1999494 (PMC8765243; doi:10.1080/07853890.2021.1999494)
Supplement: Supplemental Material [file IANN_A_1999494_SM9330.zip › Supplemental files/Table S1 Revised.doc]

**Table S1. Search strategies.**

| **Database** | **Period of Search** | **Search strategies** |
| --- | --- | --- |
| PubMed | August 2021 | (((((((((("pulmonary disease, chronic obstructive"[MeSH Terms] OR ("pulmonary"[All Fields] AND "disease"[All Fields] AND "chronic"[All Fields] AND "obstructive"[All Fields]) OR "chronic obstructive pulmonary disease"[All Fields] OR ("chronic"[All Fields] AND "obstructive"[All Fields] AND "pulmonary"[All Fields] AND "disease"[All Fields] AND "copd"[All Fields]) OR "chronic obstructive pulmonary disease copd"[All Fields]) AND (("lung"[MeSH Terms] OR "lung"[All Fields] OR "pulmonary"[All Fields]) AND ("rehabilitant"[All Fields] OR "rehabilitants"[All Fields] OR "rehabilitate"[All Fields] OR "rehabilitated"[All Fields] OR "rehabilitates"[All Fields] OR "rehabilitating"[All Fields] OR "rehabilitation"[MeSH Terms] OR "rehabilitation"[All Fields] OR "rehabilitations"[All Fields] OR "rehabilitative"[All Fields] OR "rehabilitation"[MeSH Subheading] OR "rehabilitation s"[All Fields] OR "rehabilitational"[All Fields] OR "rehabilitator"[All Fields] OR "rehabilitators"[All Fields]))) AND ("exercise"[MeSH Terms] OR "exercise"[All Fields] OR ("exercise"[All Fields] OR "training"[All Fields]) OR "exercise training"[All Fields])) OR ("yoga"[MeSH Terms] OR "yoga"[All Fields])) OR ("tai ji"[MeSH Terms] OR ("tai"[All Fields] AND "ji"[All Fields]) OR "tai ji"[All Fields] OR ("tai"[All Fields] OR "chi"[All Fields]) OR "tai chi"[All Fields])) AND (("randomized controlled trial"[Publication Type] OR "randomized controlled trials as topic"[MeSH Terms] OR "randomized controlled trial"[All Fields] OR "randomised controlled trial"[All Fields]) AND "RCT"[All Fields]) |
| Embase | August 2021 | (exercise:ab,ti AND training:ab,ti OR yoga:ab,ti OR 'tai chi':ab,ti) AND 'pulmonary rehabilitation':ab,ti AND 'chronic obstructive pulmonary disease':ti |
| Cochrane Library | August 2021 | ((chronic obstructive pulmonary disease) OR MeSH descriptor chronic obstructive pulmonary disease explode all trees) AND (exercise training OR yoga OR tai chi) AND pulmonary rehabilitation |
